# Zebrafish as an Emerging Model Organism to Study Angiogenesis in Development and Regeneration

**DOI:** 10.3389/fphys.2016.00056

**Published:** 2016-03-08

**Authors:** Myra N. Chávez, Geraldine Aedo, Fernando A. Fierro, Miguel L. Allende, José T. Egaña

**Affiliations:** ^1^Department of Plastic Surgery and Hand Surgery, University Hospital rechts der Isar, Technische Universität MünchenMunich, Germany; ^2^Department of Biology, FONDAP Center for Genome Regulation, Faculty of Science, Universidad de ChileSantiago, Chile; ^3^Department of Biochemistry and Molecular Biology, FONDAP Advanced Center for Chronic Diseases (ACCDiS) and Center for Molecular Studies of the Cell (CEMC), Faculty of Chemical and Pharmaceutical Sciences, Faculty of Medicine, University of ChileSantiago, Chile; ^4^Department of Cell Biology and Human Anatomy, University of CaliforniaDavis, Sacramento, CA, USA; ^5^Institute for Medical and Biological Engineering, Schools of Engineering, Biological Sciences and Medicine, Pontifícia Universidad Católica de ChileSantiago, Chile

**Keywords:** *Danio rerio*, vascular development, vessel regeneration, angiogenesis assay, high-throughput screening assays, endothelial markers

## Abstract

Angiogenesis is the process through which new blood vessels are formed from preexisting ones and plays a critical role in several conditions including embryonic development, tissue repair and disease. Moreover, enhanced therapeutic angiogenesis is a major goal in the field of regenerative medicine and efficient vascularization of artificial tissues and organs is one of the main hindrances in the implementation of tissue engineering approaches, while, on the other hand, inhibition of angiogenesis is a key therapeutic target to inhibit for instance tumor growth. During the last decades, the understanding of cellular and molecular mechanisms involved in this process has been matter of intense research. In this regard, several *in vitro* and *in vivo* models have been established to visualize and study migration of endothelial progenitor cells, formation of endothelial tubules and the generation of new vascular networks, while assessing the conditions and treatments that either promote or inhibit such processes. In this review, we address and compare the most commonly used experimental models to study angiogenesis *in vitro* and *in vivo*. In particular, we focus on the implementation of the zebrafish (*Danio rerio*) as a model to study angiogenesis and discuss the advantages and not yet explored possibilities of its use as model organism.

## Introduction

Angiogenesis is the process through which new blood vessels emanate from preexisting vascular structures. It plays a pivotal role in various physiological and pathological conditions and is orchestrated by the tight interaction between endothelial cells and their niche. While inadequate vessel maintenance or growth leads to tissue ischemia; excessive vascular growth or abnormal remodeling promotes cancer, inflammatory disorders, and retinopathies (Pandya et al., 2006).

Angiogenesis is mainly accomplished through vessel sprouting, which may be divided into four main steps: tip cell formation, tubule morphogenesis and lumen creation, adaptation to tissue needs and, finally, stabilization and maturation of the newly formed vessels (Ribatti and Crivellato, 2012; Neufeld et al., 2014). A non-sprouting mechanism of microvascular growth has been also described, and it involves the increment of vascular surface by insertion of a multitude of transcapillary pillars in a process called “intussusception” (Styp-Rekowska et al., 2011).

Parallel to the study of the angiogenic process, a large number of *in vitro* and *in vivo* assays have been developed to study the cellular and molecular mechanisms involved (Cimpean et al., 2011). Each model has its own advantages and disadvantages, and their adequate combination is key to reveal the impact of the element under analysis within the global process.

*In vitro* assays have been broadly used to answer questions related to specific behaviors of endothelial cells such as proliferation, differentiation, structural organization, cytokine secretion profiling and chemotaxis, as well as the molecular mechanisms associated with angiogenesis (Irvin et al., 2014). Moreover, *in vitro* systems have helped to identify and validate promising compounds to therapeutically promote or inhibit angiogenesis (Goodwin, 2007), as they are quantitative, easily monitored, reproducible, and provide the confidence necessary for the rapid screening of potential pro- or anti-angiogenic compounds (Weiss et al., 2015). However, important aspects should be considered when assessing the potential of an angiogenic effector using *in vitro* assays such as the decision over the type or tissue-origin of the endothelial cells being used, and the experimental bias of the protocols being followed (for a more comprehensive discussion see Unger et al., 2002; Staton et al., 2009). Finally, common *in vitro* experiments do not consider the influence of the vascular niche, which has been shown to be critical in the process of angiogenesis during tissue regeneration (Ribatti and Crivellato, 2012; Kunisaki and Frenette, 2014; Ramasamy et al., 2015).

The complexities of the formation, function and pathology of blood vessels in the context of the living animal mandate the availability of adequate *in vivo* models in order to confirm the results obtained *in vitro*. Since the 1970s multiple animal models have been developed in order to understand the physiological mechanisms of blood vessel formation, as well as to validate approaches that either enhance or inhibit the angiogenic process. The mouse model is by far the most common used to study angiogenesis *in vivo*, with the advantage of being a mammal that in many ways faithfully recapitulates human physiology. However, this animal model can be laborious and expensive to use, especially for screening purposes. Also, the use of mice limits the evaluation of the outcome to a final time point, since *de novo* or re-vascularization can only be visualized and quantified after euthanizing the animal, hence limiting the understanding of angiogenic dynamics.

As mammalian and most vertebrate tissues are opaque, the introduction of the transparent zebrafish larva as a tool for the examination of the vasculature in the intact animal has gained recent attention. Importantly, several studies have made clear that there is a high degree of molecular conservation in the most important pathways involved in the development and physiology of blood vessels in all vertebrates (reviewed by Baldessari and Mione, 2008; Gore et al., 2012). Furthermore, genetic and pharmacological evidence has shown that there is mutual translatability of findings between zebrafish and human vascular biology (Coultas et al., 2006; Lieschke and Currie, 2007). Thus, the emergence of a simple yet validated discovery and/or screening tool has been welcomed by the community.

In the following sections, we provide a brief overview on the currently available *in vitro* and *in vivo* angiogenic assays, describing their most common uses and their potential advantages and limitations. Additionally, we also provide information on the current and potential uses of zebrafish as model to study angiogenesis.

## *In vitro* models

*In vitro* angiogenesis models study the behavior of endothelial cells within a controlled environment (Ayata et al., 2015). They are designed to recapitulate the different steps of the angiogenic processes, where endothelial cells are involved, such as cell proliferation, migration, extracellular matrix digestion and invasion, morphogenesis and capillary tube formation (Cimpean et al., 2011). Table 1 summarizes the settings and evaluation parameters of the most commonly used assays focusing on migration, proliferation and tubule formation.

**Table 1:** ***In vitro* angiogenesis assays**.

**Type of assay**	**Basis**	**Assay**	**Setting**	**References**
Proliferation (reviewed by Stoddart, 2011; Niles and Riss, 2015)	Cell number		The effect of test substance is measured by estimation of the increase in viable endothelial cell number over time	Staton et al., 2009
	Cell cycle kinetics	BrdU assay	Bromodeoxyuridine (BrdU), a pyrimidine analog, is incorporated during DNA synthesis and quantified by immunohistochemistry or ELISA	Qin et al., 2006
		Proliferation marker detection assay	Ki-67, expressed during the S, G2 and M phases, or the proliferating cell nuclear antigen (PCNA), overexpressed in the G1 and S-phase are estimated quantitatively	Whitfield et al., 2006
	Metabolism	Tetrazolium salt-assays	Metabolically active cells convert tetrazolium-salt compounds (MTT, XTT, MTS and WST1) into formazan dyes. The colorimetric change is quantified using spectrophotometry and correlated to cell number	Boncler et al., 2014
		Protease activity assay	Protease activity measured using a fluorogenic cell permeable substrate (glycyl-phenylalanyl-aminofluorocoumarin; GF-AFC) is correlated to viable cell-number	Niles et al., 2007
		Resazurin assay:	Metabolically active cells reduce resazurin to resorufin, changing the spectrometric properties of the compound. Signal is quantified and correlated with cell number	Larson et al., 1997
		ATP-measurement	Bioluminescence-based ATP-detection assay that uses the linear relationship between viable cell number and ATP-concentration	Wang et al., 2010
	Cell death	TUNEL-assay	Fluorescent labeling of terminal deoxynucleotidyl transferase-dUTP nick end of the 3′-OH region of fragmented DNA is estimated by microscopy or flow cytometry	Goodwin, 2007
		Apoptosis marker detection assay	Expression of apoptosis cell-markers, such as caspase-3 or annexin V, is assessed via microscopy or flow cytometry	Köhler et al., 2002
		LDH assay:	The release of lactate dehyrogenase (LDH) as a consequence of loss of cell membrane integrity can be quantified to through a colorimetric reaction	Smith et al., 2011
Migration (reviewed by Hulkower and Herber, 2011)	Wound assay	Scratch assay	A tip or needle is used to remove cells to form a denuded area in a confluent endothelial cell monolayer, in which cell migration can be quantitatively estimated after a specific time interval	Steinritz et al., 2015
		Exclusion zone assay	Stencils are placed in culture plates prior to cell-seeding in order to create uniformly sized wounds in an intact confluent monolayer, in which invasion by the patterned cells can be quantitatively assessed	Gough et al., 2011
	Chemotaxis/ chemoinvasion	Boyden chamber assay	Two-compartment chamber with a semi-permeable membrane is used to evaluate active cell migration in response to specific stimuli or due to chemotaxis within a test substance gradient	Albini and Benelli, 2007
		Microfluidics assay	Creation of a diffusion-generated concentration gradient within a migration chamber, through which endothelial cells can migrate	Chung et al., 2010; Young, 2014
Morphogenesis (reviewed by Arnaoutova and Kleinman, 2010)	Tubule formation	2D-tubule formation assay	Endothelial cells are platelet on an extracellular matrix and monitored for their ability to form vessel-like tubules	Arnaoutova and Kleinman, 2010
		EC-aggregate reassembling assay	Endothelial cell spheroids or aggregates are embedded in an extracellular matrix that resembles the basement membrane environment. Upon stimulation, vessels sprout into the matrix	Li and Stuhlmann, 2011
		3D-tubule formation assay	Endothelial cells are seeded in a three-dimensional culture platform that involves extracellular matrix components and/or other cell-types. Different settings allow to study sprouting, formation, stabilization and maturation of vessel-like tubules	Hetheridge et al., 2011; Diaz-Santana et al., 2015

### Proliferation assays

These assays are conceived to evaluate the effects of a test substance, based on the quantitation of endothelial cell proliferation. They are broadly classified into those that determine net cell number and those that evaluate cell-cycle kinetics (Staton et al., 2004). Cell numbers can be estimated either manually or through automated cell counting. Alternatively, metabolic assays, which have shown a linear correlation with cell density (Niles and Riss, 2015), quantification of DNA synthesis or expression of proliferation markers may be used (reviewed by Whitfield et al., 2006). However, since none of these methods have been explicitly developed for vascular-related cells, it is indispensable to address the target specificity of the test substance, as well as its therapeutic impact based on other angiogenesis-related parameters. Furthermore, proliferation assays should be combined with quantitative methods for estimation of cell death, in order to discard the possibility of cytotoxicity of the test-substance (Kepp et al., 2011).

### Migration assays

Migration assays allow the study of endothelial cell motility and chemotaxis. They evaluate the active migration of cells into a specific area or toward a specific direction as a result of a treatment. The main advantage of the exclusion zone assay (Poujade et al., 2007; Gough et al., 2011), where silicone-based structures, so-called “masks” or “stencils,” are placed on the well bottom to create an cell-empty area, in comparison to a scratch assay (Coomber and Gotlieb, 1990; Yarrow et al., 2004), where a “wound” is created by physically disrupting an endothelial cell monolayer, is the uniformity and hence reproducibility of the denuded area into which confluent endothelial cells will later migrate (reviewed by Hulkower and Herber, 2011).

Another commonly used migration assay follows the principle of the Boyden chamber, first described in 1962, where a semipermeable membrane that only allows active passage of cells is placed in their migration path (Boyden, 1962), sometimes requiring matrix degradation, in which case it is regarded a chemoinvasion assay (Albini and Benelli, 2007), or in response to a test substance (chemotaxis). The use of microfluidic cell culture systems has overcome the difficulties of maintaining a linear gradient of the test compound by introducing microchannel compartments in which a diffusion-generated concentration gradient can be created. Furthermore, they allow live single-cell and cell-population tracking, as well as directionality and velocity estimation (Young, 2014).

### Tubule formation assays

Tubule formation assays are used to study the assembly of capillary-like structures by adjacent endothelial cells (Arnaoutova et al., 2009). In two-dimensional assays, endothelial cells are usually seeded on extracellular matrices and the spontaneous building of capillary-like networks is analyzed. Quantitation of tubule formation is mainly addressed by immunohistochemistry and analyzed based on four main parameters: average tubule length, number of tubules, tubule area and number of branch points (Staton et al., 2009). They allow to study spontaneous tubule formation due to endothelial cell-to-cell interactions and the assembly of tight-junctions (Vailhé et al., 2001), however they do not resemble the process of sprouting angiogenesis, which is the development of new blood vessels from pre-existing major donor vessels (Ribatti and Crivellato, 2012). Also, the early formed tubules lack lumen and their length and degree of branching differ from real capillaries (Donovan et al., 2001).

On the other hand, three-dimensional culture systems of endothelial cells have been used to study the formation of more complex capillary networks inside extracellular matrix substitutes. They have helped to elucidate the role of support cells, such as fibroblasts (Bishop et al., 1999; Hetheridge et al., 2011), pericytes (Berthod et al., 2012) and adipose stromal cells (Merfeld-Clauss et al., 2010; Verseijden et al., 2010; Sarkanen et al., 2012), as well as the homo- and heterotypic cell-interactions of endothelial cells during vessel-formation, -sprouting and -anastomosis (Ayata et al., 2015; Diaz-Santana et al., 2015). Moreover, three-dimensional tubule formation assays have become an important tool to mimic *in vitro* microenvironments of tumor vascularization (reviewed by Chwalek et al., 2014; Song et al., 2014). Low standardized settings and the more challenging evaluation of the three-dimensional tubule formation are the main disadvantages of these assays.

### Organ explant based-assays

Also known as *ex vivo* angiogenesis models, these assays aim to analyze the angiogenic sprouting and the growth of vessel capillaries from explanted segments of vasculature. Here, isolated vasculature biopsies are placed generally over three dimensional biological matrices in the presence or absence of a test compound. Explants are then monitored for the outgrowth of vessel tubules extending from the periphery of the explant into the surrounding matrix (Rezzola et al., 2014). Table 2 summarizes the characteristics of the most broadly used *ex vivo* assays.

**Table 2:** ***Ex vivo* angiogenesis assays**.

**Assay**	**Setting**	**Advantage**	**References**
Rat aortic ring assay	Thoracic aorta is dissected, cleaned and cut into rings. Upon serum-starvation, rings are embedded in extracellular matrix components in the presence or absence of the test compound. Exponential vessel outgrowth from the explant of the tubule structures is observed within 10 days	Many rings available from few animals. Supporting cells are included in the formation of vessels. Visible lumenized tubule structures develop over a time course similar to that *in vivo*	Nicosia, 2009
Mouse aortic ring assay		Cost-efficient transgenic mouse technologies and gene manipulation available. Implementable for high-quality imaging and high-throughput screening	Baker et al., 2011
Miniature ring-supported gel assay	Isolated aortae segments are placed in low volume three-dimensional collagen gel supports, which are casted by a nylon mesh ring that improves the stability of the setting	Optimized system allows better specimen handling, staining, imaging, and a more economical use of extracellular matrix reagents	Reed et al., 2011
Human arterial ring assay	Human umbilical arteries are isolated from umbilical cords, sectioned into rings, and then embedded in extracellular matrix. Tubular structures are quantified by image analysis	Provides a three-dimensional system for identification of genes and drugs that regulate human angiogenesis	Seano et al., 2013
Retinal explant assay	Explanted retina is cut and placed, over a three-dimensional gel with the photoreceptor layer facing upward. Endothelial cell sprouting is observed from day 3 and peaks at day 7	Allows the study of tip endothelial cell angiogenic responses and acute responses of retinal blood vessels at the sprouting front	Rezzola et al., 2014
Fat-tissue microfragment assay	Human subcutaneous fat tissue is fragmented and embedded in fibrin. Blood vessel growth and elongation is examined after 15 days by microscopy	Uses intact human fat tissue with quiescent vessels from which other spontaneously derive. Assay could help predict response toward a treatment	Greenway et al., 2007
Choroid sprouting assay	The choroid, a vascular bed beneath the retinal pigment epithelium, is separated from the retina, segmented, and placed over a matrix. Outgrowth of vascular sprouts can be observed within 2-6 days.	Vascular sprouts consist of endothelial cells, pericytes and macrophages. Robust, reproducible and representative model of microvascular angiogenesis Semi-automated software for quantification of sprouting area is available	Shao et al., 2013

*Ex vivo* assays have the advantage of working with native quiescent endothelial cells *in vivo* at the experimental outset (Ucuzian and Greisler, 2007; Staton et al., 2009). Further, because the tissue complexity is preserved, most of the cellular and molecular components involved in angiogenesis are present. As a result, vascular sprouts contain a lumen and a basement membrane, and are composed of a mixed population of endothelial cells, pericytes, fibroblasts, and macrophages (Nicosia et al., 2011). These assays allow the study cell proliferation, migration, tube formation, network branching, perivascular recruitment and vascular remodeling (Baker et al., 2011), in addition to other post-angiogenic mechanisms such as vessel stabilization and regression (Nicosia et al., 2011). Some of the disadvantages compared to *in vitro* assays are the more demanding technical skills, the limited number of simultaneous samples being processed, and the implicit higher experimental variability (Staton et al., 2009; Rezzola et al., 2014). On the other hand, compared to *in vivo* assays, *ex vivo* assays do not consider circulating endothelial progenitors recruited in the angiogenic process and lack the pro-angiogenic stimuli in blood flow (Irvin et al., 2014). Also, the decision over the source of the vascular material should behold that angiogenesis mainly involves the microvasculature rather than the macrovasculature, and that microvessels such as capillaries, small arterioles and venules, are composed of different tissue layers compared to large arteries and veins (Staton et al., 2009).

## *In vivo* models

Multiple *in vivo* models have been developed to directly study angiogenesis within an organism, and therefore evaluate the entire process of new blood vessel formation, since they allow to consider all cellular and molecular role players involved, such as supporting cells (e.g., tumor cells, pericytes, smooth muscle cells, and fibroblasts), the extracellular matrix, and the cellular and humoral components in circulating blood (Staton et al., 2004). Most *in vivo* angiogenesis assays are not designed to understand a specific process, but rather to determine the success of the outcome, with the exception being the zebrafish larva, as we discuss in the next section. Nevertheless, it is also important to point out that one of the main disadvantages of *in vivo* models is the ethical concerns they raise, and the complications they imply, due to the strict guidelines regulating animal testing in some countries. Again, the zebrafish is exempt, for the most part, from these concerns, especially during larval stages.

### Corneal angiogenesis assays

As originally developed by Gimbrone et al. (1973), induction of angiogenesis in the cornea is among the most convincing demonstrations of neovascularization, since the cornea is richly innervated, but normally has no blood vessels (Henkind, 1978). In this assay, a stimulus induces the migration of endothelial cells from the edge of the cornea into the space between the corneal epithelium and stromal cells, forming new sprouts directed toward the source of the angiogenic signal. This method has been applied in multiple animal models including rabbit, mouse, rat and guinea pig (Ziche and Morbidelli, 2015). It has been further developed to become quantitative, by incorporation of a contrast-dye such as high molecular weight dextran and imaging analysis. Disadvantages are that it is rather expensive, and that the angiogenic process is rather atypical, since it occurs in a non-vascular environment (Norrby, 2006).

### Chorioallantoic membrane (CAM) assay

The CAM assay allows the measurement of both inhibition and stimulation of angiogenesis over the vascularized chorioallantoic membrane of a chick embryo, which can develop normally after carefully opening the egg shell to create a window (*in ovo*), or being placed in a cup outside of the egg shell (*ex ovo* or *in vitro*), in order to get access to the CAM. From days 3.5 to 10 after fertilization, highly proliferative and immature endothelial cells rapidly grow a sprouting vascular network, which is then replaced by intussusceptive microvasculature (Ribatti et al., 2001). During early phases, the CAM assay is most suitable to study angiogenic inhibitors. In contrast, the study of pro-angiogenic factors is best accomplished from day 6 to 8, when the rapid embryonic angiogenic development has slowed down. Quantification of angiogenesis is typically based on the directionality of the blood vessels toward the graft/angiogenic stimuli, the number of sprouts, and/or the size/length of the stimulated blood vessels. The CAM assay allows repeated visualizations of the angiogenic process, and it is fast and cost effective, making it suitable for large scale screens. Its major disadvantages are the rather challenging quantification of the outcome, since it is often difficult to distinguish normal angiogenesis from the induced one, and the false positive effects that often occur from inadvertently damaging the CAM (Ribatti et al., 2001; Ribatti, 2008).

### Matrigel plug assay

Subcutaneous injection of matrigel in mice is a common method to study angiogenesis *in vivo* in mammals. Matrigel is an extract of the Engelbreth–Holm–Swarm tumor, mostly composed by extracellular matrix proteins and growth factors (Benton et al., 2014). When cold, matrigel is liquid, but becomes solid at body temperature. This property makes simple the injection of matrigel in the midventral abdominal region of mice, where it quickly solidifies forming a “plug” (Akhtar et al., 2002). The injected matrigel can be supplemented with either angiogenic inhibitors or inducers. Then, usually about 2 weeks after injection, infiltration of new blood vessels is determined histologically. A major advantage of this method, is the simplicity to implement it. However, visualization and quantification of differences can be challenging and are mainly based in the histological analysis of explanted plugs at a final experimental point.

### Hind limb ischemia

A common system to study angiogenesis *in vivo* from a therapeutic perspective, is the hind limb ischemia (HLI) model (Limbourg et al., 2009). In this case, the femoral artery of mice is ligated causing a strong obstruction of blood flow toward the hind limb. Since originally described (Couffinhal et al., 1998), the HLI protocol has been applied with multiple variations. A common surgical approach is the ligation of the femoral artery at distal and proximal sites, and removal of the intervening arterial fragment (Fierro et al., 2011). Another approach is a single ligation, without arterial excision, where the severity of ischemia depends on the specific site of ligation. Also a gradual arterial occlusion model has been established, by placing ameroid constrictors on the femoral artery (Yang et al., 2008). In all cases, the contralateral hind limb is left intact, as a control. Mice are usually able to recover from this injury naturally, restoring blood flow within approximately 4 weeks, by mechanisms including the formation or enlargement of collateral blood vessels (Sondergaard et al., 2010). Laser scanning Doppler imaging is the best suited method to monitor blood flow restoration upon HLI induction, because it is non-invasive, and can be performed in the same animal at multiple time points. At the end of the experiment, animals can be euthanized for further investigation including histology and gene expression analysis. A negative aspect of scanning Doppler imaging is the sensitivity of the method, since only robust differences can be noticed. Another limitation of this method is that it fails to reveal the exact mechanism underlying the blood flow restoration (e.g., angiogenesis vs. vasculogenesis).

### Vascularization during dermal wound repair

Our group has developed a full skin defect model that presents several advantages compared to the *in vivo* models presented above, which are intrinsic to the nature of skin. Among others: transparency, large surface, easy manipulation, external location and tissue homogeneity (Egaña et al., 2008). In this model, full skin defects are surgically created bilaterally on the back of mice, and the skin excision is replaced by biodegradable scaffolds, which can be modified to contain a specific angiogenic stimuli. Typically, after two weeks animals are euthanized, and tissue vascularization is quantified as follows: the skin, including the implanted scaffold, is removed and quickly placed over a light source. During trans-illumination, a digital picture is taken, and is later analyzed by digital segmentation (Schenck et al., 2014). This method does not affect cell integrity post mortem, allowing further analysis such as histology or protein/RNA extraction.

### The skinfold chamber and ear assays

Four major types of *in vivo* models have been developed to observe the angiogenic process in two dimensions: the rat mesentery window assay (Norrby, 2011), the hamster cheek pouch assay (Monti-Hughes et al., 2015), the dorsal skinfold chamber adapted to mice, hamsters and rats (Lehr et al., 1993; Harder et al., 2014; Irvin et al., 2014), and the rabbit ear chamber assay (Clark et al., 1931; Ichioka et al., 1997). These techniques, developed as early as in the 1940s, rely on semi-transparent tissue or the implantation of a transparent chamber that allows an easy and direct visualization and quantification of the angiogenic process, including blood vessel density and blood flow velocity. In particular, the implementation of intravital microscopy along with epifluorescence, confocal and multiphoton techniques, offers the possibility of repetitive, direct, and quantitative measurements of several microcirculatory parameters, as well as microvasculature imaging at an unparalleled subcellular-resolution (Taqueti and Jaffer, 2013). However, these methods are invasive, and may cause great discomfort to animals. In addition, some methods such as the implantation of a dorsal window chamber in mice, are cumbersome (Palmer et al., 2011) and therefore difficult to implement in a number of animals sufficient for adequate technical replicates.

## Zebrafish as a model for angiogenesis research

While the models described above have provided essential information and platforms for discovery of therapeutic targets and drugs, many questions about the biology of vascular cells and how they build the circulatory system remain unresolved. Above all, the relevance of the models is often hindered by the inaccessibility of the tissue in live animals, and much of what we know has been derived from fixed material or indirect assays. Zebrafish provides a series of advantages as a model of study due to its rapid development, optical transparency, high number of offspring and straightforward strategies for forward and reverse genetic manipulation. Furthermore, the early development of a cardiovascular system in the transparent zebrafish embryo and larva translates into a unique opportunity for direct observation of blood flow and the development of the system's related organs in both wild type and transgenic fish, without the need for complex instrumentation. Lastly, genetic studies have revealed conservation of the molecular pathways between fish and mammals making research in vascular biology in teleosts directly translatable into potentially relevant information for human health.

As the restrictions on the experimental use of mammalian models for research increase, the zebrafish emerges as a convenient alternative. Larvae can be used in massive numbers in genetic or pharmacological screens, at stages in which they lack the legal status of a “vertebrate animal” yet have all of the physiological functions of the adult, including a hematovascular system. Circulation begins 24 h after fertilization, with a simple, yet functional blood circuit. The embryos and larvae, can be kept for the first five days of life in small wells in microtiter plates, in only a few hundred microliters of water. This is the pharmacologists dream since as many replicates of the experiment as one desires can be done and dilutions of each drug can be tested *ad libitum*.

Two decades ago, the generation of the first stable transgenic zebrafish line was reported. Since then, hundreds of transgenic lines have been developed both for expression of reporter proteins or for expression of diverse proteins for functional studies (Udvadia and Linney, 2003). At the same time, efficient mutagenesis protocols have allowed forward-genetic screening in the context of angiogenesis, generating valuable collections of mutants (Jin et al., 2007). Traditionally, gene function in zebrafish has been assessed using chemically or insertionally induced mutants that required large scale unbiased screens to identify phenotypes related with the process or organ of interest (Gaiano et al., 1996; Haffter et al., 1996). While common antisense technologies were not generally applicable to the zebrafish, the advent of oligonucleotide substitutes named morpholinos, enabled the knockdown of endogenous genes by either blocking translation of the mRNA or splicing of the pre-mRNA (Nasevicius et al., 2000). The ease of this technology spurred its widespread use, even though it presented some limitations such as the induction of undesired off-target effects or the progressive loss of the effect at late developmental stages because of diminishing activity over time. The zebrafish toolkit has been recently enriched with the introduction of gene editing technologies such as TALENs (Transcription activator-like effector nucleases, Bedell et al., 2012), and CRISPR (Clustered regularly-interspaced short palindromic repeats)-Cas based strategies (Hwang et al., 2013). As long as genomic sequence is available for the targeted locus, any gene can be mutated efficiently and permanently in the germ line; the efficiency is often high enough such that recessive phenotypes can be seen already in the injected animals. Further, the CRISPR-Cas9 system has been adapted for high throughput mutagenesis in zebrafish so that dozens of genes can be mutated in a single experiment (Varshney et al., 2015). Recently, phenotypic inconsistencies between genomic mutations induced by CRSPR-Cas9 and knockdown via morpholinos have emerged (Kok et al., 2015). It is likely that these two gene loss-of-function strategies differ in their penetrance given that genetic lesions might induce compensatory reactions in the genome obscuring the gene's function. Many authors believe that a combination of strategies is desirable when analyzing a particular gene and that it is unwise (as has been agreed by communities using other model organisms) to rely only on a gene knockdown phenotype to assign gene function (Lawson, 2016).

Despite its success and popularity, those working with the zebrafish model must consider complementing their studies with mammalian systems, if they wish to validate the knowledge gained for potential clinical applications. Gene and protein functional conservation is high, but not absolute, and obviously there are important physiological differences to be dealt with. Aquatic and terrestrial life pose unique challenges that impact on many organs, most notably the respiratory system and, thus, cardiovascular architecture. In fish, only the embryo and larva are transparent, making studies in adults just as difficult as in mammals. The small size of embryos makes some observations challenging (i.e., requiring sophisticated microscopy and imaging) and they are also developing systems, which means they are constantly in a state of change and growth. Thus, the zebrafish, with all of its attributes, should be considered a starting point for discovery and a model that can offer new hypotheses to be tested further in other models.

### Vascular development in zebrafish

Transgenic technology has enhanced the inherent *in vivo* imaging capabilities that zebrafish larvae may offer to the investigator. Though vessels and blood flow can easily be visualized with a simple dissecting scope, it was with the introduction of tissue specific expression of fluorescent proteins that vascular and blood development could be examined in great detail. Confocal microscopy and time lapse imaging can both be carried out with live specimens which allows detailed morphogenetic movements and cell shape changes to be followed directly. Thus, vascular development has been described in great detail, both from the anatomical and cellular point of view and with a comprehensive examination of the molecular players involved (reviewed by Gore et al., 2012; Schuermann et al., 2014).

Most of the strategies which have been followed to create stable transgenic lines with vascular-specific phenotypes are based on gene-specific promoters. Both autologous and heterologous promoters have been shown to work. Table 3 lists some of the transgenic lines, which have been designed and developed for the visualization and analysis of the vascular system. Before a complete and reliable zebrafish genome sequence was available, the promoter of a related gene from another species, most commonly a mammalian one (Baldessari and Mione, 2008), was used. However, the reporter protein expression in zebrafish did not always exactly recapitulate that of the orthologous one, because of the differences in promoter elements among species. For example, the zebrafish Tg(tie2:GFP)s849 line encoding the promoter for the murine tie2-gene (a vascular-specific tyrosine kinase receptor activated by angiopoietin ligands), successfully drove GFP expression in endothelial cells, but also showed substantial nonvascular expression in the hindbrain and the posterior neural tube, and the overall level of expression was proportionally lower compared to that in mice (Motoike et al., 2000). On the other hand, the fli1a and scl zebrafish genes, have been used as early markers of vascular and hematopoietic lateral mesoderm. While the expression of fli1a is restricted to endothelial cells, a subset of early circulating myeloid cells, and cranial neural crest derivatives (Brown et al., 2000), the expression of scl is specific for the hematopoietic lineage at later stages (Gering et al., 1998).

**Table 3:** **Transgenic zebrafish lines generated for the study and visualization of the vascular system**.

**Line**	**Gene**	**Expression**	**References**
*Tg(5xUAS:cdh5-EGFP)*	*VE-cadherin*	Pan- endothelial	Lenard et al., 2013
*Tg(-7.8gata4:GFP)ae3*	*Transcription factor GATA-4*	Endocardial and myocardial cells	Heicklen-Klein and Evans, 2004
*Tg(dll4:EGFP)*	*Notch ligand*	Endothelial cells	Sacilotto et al., 2013
*Tg(efnb2a:EGFP)*	*Ligand of Eph- receptor*	Artery	Swift et al., 2014
*Tg(fli:eGFP)y1*	*Transcription factor Fli-1*	Endothelial cells, cytoplasmic	Lawson and Weinstein, 2002a
*Tg(fli1:neGFP)y7*	*Transcription factor Fli-1*	Endothelial cells, nuclear	Roman et al., 2002
*Tg(flt4:YFP)*	*Vegfr3*	Pan-endothelial	Hogan et al., 2010
*Tg(gata1:dsRed)sd2*	*Transcription factor GATA-1*	Blood cells	Traver et al., 2003
*Tg(gata1:GFP)*	*Transcription factor GATA-1*	Erythroid lineage	Long et al., 1997
*Tg(gata2:eGFP)*	*Transcription factor GATA-2*	Blood cells	Traver et al., 2003
*Tg(hsp70l:canotch3-EGFP)*	*Notch3 intracellular domain*	Perivascular	Wang et al., 2014
*Tg(kdr.eGFP)s843*	*Vegfr2/flk1/kdr/Vegfr4*	Angioblast/endothelial precursors	Jin et al., 2006
*Tg(kdr:G-RCFP)*	*Vegfr2/flk1/kdr*	Angioblast/endothelial precursors	Cross et al., 2003
*Tg(kdr:RFP)la4*	*Vegfr2/flk1/kdr*	Angioblast/endothelial precursors	Huang et al., 2005
*Tg(my17:eGFP)*	*Cardiac myosin light chain 2*	Myocardial cells	Ho et al., 2007
*Tg(nkx2.3:efnb2a,myl7:EGFP)*	*Ligand of Eph- receptor*	Artery	Choe and Crump, 2015
*Tg(scl-α:DsRed)*	*Transcription factor Tal-1*	Endothelial cells (intermediate)	Zhen et al., 2013
*Tg(scl-β:d2eGFP)*	*Transcription factor Tal-1*	Endothelial cells (anterior-posterior)	Zhen et al., 2013
*Tg(Tie2:eGFP)*	*Tie-2 receptor tyrosine kinase*	Endothelial cells	Motoike et al., 2000
*TgBAC(cdh5:Citrine)*	*VE-cadherin*	Pan- endothelial	Bussmann and Schulte-Merker, 2011
*TgBAC(cdh5:GAL4FF)*	*VE-cadherin*	Pan- endothelial	Bussmann et al., 2011
*TgBAC(dll4:GAL4FF)*	*Notch ligand*	Endothelial cells	Hermkens et al., 2015
*TgBAC(flt4:Citrine)*	*Vegfr3*	Pan-endothelial	Gordon et al., 2013
*Tg(0.8flt1:RFP)hu5333*	*Flt1*	Strong expression in arterial ISV	Bussmann et al., 2011

*Adapted from Baldessari and Mione (2008), Kamei et al. (2010) and Schuermann et al. (2014)*.

The development of the vascular anatomy of the zebrafish has been extensively described and has been proven to share high similarity with other vertebrates (Isogai et al., 2001; Ellertsdóttir et al., 2010; Gore et al., 2012). Many of the studies on vascular development have been achieved by using molecular tracers during the early embryonic stages of zebrafish. One of such strategies is the injection of fluorescent microspheres, and their detection after lumenization and anastomosis of the vascular network is complete (Küchler et al., 2006). This strategy has also been used to compare the development of blood and lymphatic vasculature in zebrafish (Coffindaffer-Wilson et al., 2011). Transgenic zebrafish lines have been also employed to track individual cell growth during vascular development. Using fluorescent endothelial cell markers, it is possible to observe the proliferative and migratory behaviors of single cells, and different kinds of cell types during the embryo-to-larva transition. Combining transgenic lines expressing different fluorescent proteins, it was possible to observe two cell types simultaneously. For instance, it was possible to track both endothelial progenitors and erythrocytes while following the vascular network development and the initiation of blood circulation (Lawson and Weinstein, 2002a,b; Herwig et al., 2011; Kimura et al., 2013). Moreover, combining nuclear and cell membrane specific fluorescent tags has allowed the examination of single cell morphological dynamics in living larvae during vessel formation (Yu et al., 2015). Finally, the development of stable transgenic zebrafish lines has been a valuable resource for tissue specific gene expression as well as inducible gene expression (Udvadia and Linney, 2003). The implementation of these strategies enabled the study of the sequence of events involved in the establishment of the first circulatory loop in zebrafish embryos, which consists in the connection between the heart with the dorsal aorta and the cardinal posterior vein back to the heart. Other blood vessels, which are characteristic and highly accessible in the zebrafish embryos and larvae are the intersegmental vessels, which emerge from the dorsal aorta into the embryonic trunk and tail, and later grow into the anastomosing dorsal longitudinal vessels (Strilić et al., 2009).

A remarkable feature of zebrafish compared to other vertebrates, is that they rely on passive oxygen diffusion during the early embryonic stages rather than oxygen perfusion, as the completion of the vascular development takes place after hatching. Moreover, the generation and characterization of zebrafish mutants has shown that embryos are able to sustain normal development even in absence of a functional vascular system or in the absence of blood (Stainier et al., 1995; Isogai et al., 2003). This attribute has made the analysis of late phenotypes related to circulatory system malformations possible, whereas they are lethal and hence impossible to study in living mammals (reviewed by Isogai et al., 2001; Wilkinson and van Eeden, 2014). A prime example of the power of the genetic approach was the study of the zebrafish *gridlock* mutant (Peterson et al., 2004). The *gridlock* mutation causes a syndrome similar to human aortic coarctation disrupting blood flow in the aorta. Further, mutant animals were used to design a small molecule screen that would detect reversal of the phenotype upon treatment and several compounds were found to have such an effect. Table 4 summarizes some of the most remarkable vascular zebrafish mutant lines described thus far.

**Table 4:** **Zebrafish vascular mutants**.

**Line**	**Gene**	**Phenotype**	**References**
*cloche*	*scl, lmo2, gata1, gata2, flt1, flt*	Lack endothelial and circulating blood cells	Stainier et al., 1995
*glass onion/parachute*	*cdh2*	Neuronal-cadherin (N-cadherin/Cdh2)-deficient zebrafish show dysmorphic vascular network	Bagatto et al., 2006
*gridlock*	*hey2*	Lack trunk and tail circulation due to reduced arterial gene expression and improper assembly of the dorsal and lateral aortae	Lawson et al., 2001
*heart of glass*	*heg*	Morphological cardiovascular defects	Mably et al., 2003; Kleaveland et al., 2009
*kurzschluss*	*unc45a*	Branchial arteries fail to form properly. Arterial-venous shunts lead to loss of circulation in the trunk	Chen et al., 1996
*lmo2*	*lmo2*	Abnormal ocular blood vessels cause failure of optic fissure closure	Weiss et al., 2012
*mindbomb*	*notch5*	Mutants are defective for Notch signaling, exhibit arterial-venous shunts, defective PCV formation, and reduced arterial gene expression	Lawson et al., 2002
*out-of-bounds*	*plexnD1*	Display premature sprouting and mispatterned growth of the trunk intersegmental vessels due to loss of semaphorin–plexin signaling pathway	Childs et al., 2002
*plcgy10*	*plcg1*	Deficient in VEGF-mediated angiogenesis and arterial differentiation	Lawson et al., 2003
*santa*	*ccm1*	Severe dilation of major blood vessels, followed by a thinning of cell walls	Mably et al., 2006
*schwentine*	*flk1*	Loss of angioblasts and failure to undergo angiogenesis	Habeck et al., 2002
Segmental artery mutants	*kdrl, plcg1, plexinD1, etsrp*	Vascular mutants identified by haploid transgenic screening show defects in Vegf/Plcg1 signaling	Covassin et al., 2009
*sonic you*	*shh*	Defects in trunk circulation due to abnormal arterial differentiation	Lawson et al., 2002
*stalactite*	*mtp*	Mutant shows excessive sprouting angiogenesis due to loss of apolipoprotein-B regulation	Avraham-Davidi et al., 2012
*tie2-hu1667*	*tie2*	Enhancement of junctional integrity via VE-cadherin	Gjini et al., 2011
*valentine*	*ccm2*	Altered endothelial junctional integrity causes dilation of major vessels.	Mably et al., 2006
*ve-cadherinubs8*	*cdh5*	Failure to form established junctions during anastomosis	Lenard et al., 2013
VEGF-receptor mutants	*flk1*	Mutants identified in a forward genetic screen show disrupted blood vessels sprouting of normal angioblasts	Habeck et al., 2002
*vhl*	*vhl*	Increased VEGF-signaling induces aberrant angiogenic sprouts and retinal neovascularization	van Rooijen et al., 2009
*violet beauregarde*	*alk1*	Mutants develop severe edema, associated with an abnormal blood circulation and improper arterial-venous connections	Roman et al., 2002

*Adapted from Lagendijk et al. (2014) and Wilkinson and van Eeden (2014)*.

Finally, experimental analysis of blood vessels during zebrafish development has also relied on common techniques for visualizing gene and protein expression. In order to observe the expression of endogenous genes in zebrafish embryos and larvae, two methods are available: *in situ* hybridization and immunohistochemistry. While neither of these methods was specifically developed for the zebrafish vasculature studies, an increasing number of tools and protocols are becoming available that facilitate these strategies (Kamei et al., 2010; Thisse and Thisse, 2014).

### Vascular regeneration

The zebrafish is a broadly known model for studies on tissue regeneration. In this regard, its capacity to regenerate its organs and limbs is remarkable even in adult stages. The caudal fin, in particular, provides an ideal tissue for studies related to vascular regeneration in adult zebrafish due to its simple thin architecture and relative transparency (Poss et al., 2003). While caudal fin regeneration in zebrafish larvae takes a few days, it has been demonstrated that the adult caudal fin is capable of full regeneration after successive amputations within a couple of weeks (Azevedo et al., 2011). The caudal fin amputation model has been extensively used to study the orchestration of the mechanisms involved in regeneration, such as cell differentiation, migration and patterning, which lead to the restoration of the fin's original morphology and functionality (Pfefferli and Jaźwińska, 2015). In a landmark study, Xu et al. (2014) showed that regenerating vessels in the regenerating tail fin originate from vein-derived cells that acquire angiogenic potential. These cells migrate singly or collectively and organize into vessel in response to chemokine signaling (reviewed by Hasan and Siekmann, 2015). However, the applicability of this model to the study of vascular regeneration could be much more widely exploited. For instance, the ablation of single vessels or vessel interruption has not been addressed in the zebrafish. A new technique called electroablation (Moya-Díaz et al., 2014) has been shown to be useful for inducing small tissue lesions including blood vessel ablation in the adult tail fin.

**Figure 1:**
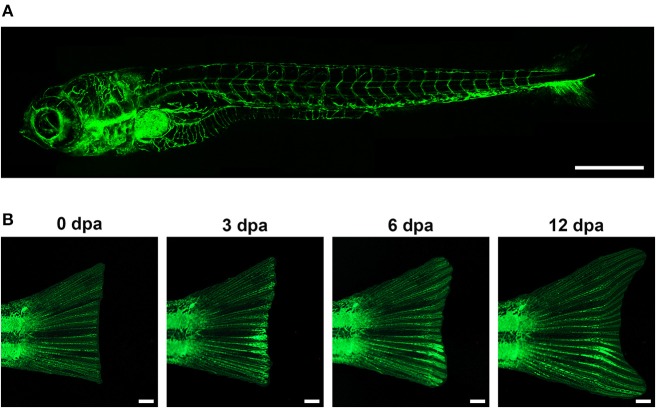
**In the transgenic zebrafish line Tg(fli1:EGFP)^*y*1^, the promoter for the endothelial marker *fli1* drives the specific expression of EGFP in blood vessels**. This allows the visualization, and hence the analysis of the vasculature during zebrafish embryonic development (**A**, Lawson and Weinstein, 2002a), and during adult vessel regeneration upon tail fin amputation (**B**, Huang et al., 2003). Scale bar represents 500 μm in **(A)**, and 1 mm in **(B)**.

The zebrafish larval vascular network has been subject of numerous screens over the past decade. Key to this effort was the development of the Tg(fli1:EGFP)y1 transgenic line (Lawson and Weinstein, 2002a), that fluorescently labels endothelial cells throughout life (Figure 1) and enables the visualization of the microvasculature in this tissue. However, most screens to date have used the larval vasculature to find molecules that disrupt (positively or negatively) the normal pattern of blood vessels. Only a few screens have examined the role of the vasculature on tissue regeneration, even though these transgenic fish could be a remarkable tool to allow the study of the effects of test substances and genetic interference on vessel growth and restoration. As an example, Bayliss et al. examined the requirement for blood vessels in caudal fin regeneration using adult fish (Bayliss et al., 2006). In this work, the authors conclude that up to ~1 mm avascular caudal fin tissue can be regenerated, though, for regeneration of the full limb, angiogenesis is required. Further, they showed that the model can be implemented for antiangiogenic drug screening, as it is possible to selectively inhibit highly active, abnormal vessels while leaving quiescent vessels intact.

Since angiogenesis is one of the main focuses of vascular regeneration research, models for this type of vascular development and growth have been developed. In the embryo, the intersegmental vessels form by angiogenic sprouting from the dorsal aorta and have been the target of studies using drugs or genetic perturbations (Schuermann et al., 2014). Further, since it has been shown that mammalian malignant cells can be xenotransplanted into zebrafish embryos and that they can form tumors (Haldi et al., 2006), models for tumor angiogenesis have been developed (Tobia et al., 2011). We have also shown recently (Chávez et al., 2016), that angiogenic sprouting can also be induced by xenotransplantation of cells expressing the recombinant vascular endothelial growth factor (VEGF), in this case plant cells. Thus, the factors governing angiogenic growth and inhibition are amenable to be examined *in vivo* in these contexts.

### High throughput screens

As previously mentioned, zebrafish larvae are optically transparent until 5 days after fertilization allowing direct observation of internal tissues. This feature, coupled with the use of transgenic zebrafish lines with fluorescently labeled organs and cells, has allowed for straightforward assays to be developed to assess either positive or negative effects of chemicals or genetic perturbations on vascular integrity (Raghunath et al., 2009; Taylor et al., 2010). For instance, by using transgenic lines in a genetic screen, numerous vascular-specific mutations were identified (Covassin et al., 2009), while a chemical screen has revealed compounds that restored a normal phenotype in mutant fish (Hill et al., 2005; Asnani and Peterson, 2014).

How relevant are drug screens carried out in fish to human biology? As most human genes have a fish ortholog and sequence conservation is high, most teleost proteins targeted by drugs will predict an effect on its human counterpart (Tran et al., 2007). The relevance of this type of approach is highlighted by the fact that several small molecules identified in zebrafish are currently in clinical trial phase (MacRae and Peterson, 2015). Furthermore, these assays can be scaled into high throughput screens due to the fact that the zebrafish larvae, 2–3 mm at 3 days post-fertilization, can be arrayed into microwell plates and examined manually or automatedly by the thousands. Large chemical libraries can be screened for direct effects on the tissue of interest as compounds readily permeate the animal, and minimal amounts of each compound are required (drugs are supplied diluted in only a few ml in aqueous solution). The readout can be exceedingly simple: usually a perturbation of the normal or expected anatomical structure or cellular behavior is sought. While it is possible to visually screen hundreds of fish for a phenotype as it has been classically done (i.e., double blind scoring), there are automated and semi-automated systems for image acquisition and analysis as well as software that can quantitatively detect subtle effects (Pardo-Martin et al., 2010; Tamplin and Zon, 2010).

## Conclusions and perspectives

Since the 1990s experimentation on animals has increasingly emphasized the “three Rs”: reduction (minimize the number of animals), refinement (maximize the amount of data obtained) and replacement, (substitute with *in vitro* studies, when possible; Mayer et al., 1994). Here, we have enumerated a series of alternative models for the study of vascular development and regeneration. *In vitro* studies are accessible and offer controlled conditions for manipulation, but they lack the complexity found in living tissues. As mammalian models present the closest substitutes for humans, they should be preferred as the final validation step when proposing a therapy. However, these organisms can only be used in small numbers due to the cost, cumbersomeness of the experimental designs and ethical concerns. We describe the zebrafish model as an attractive alternative because it combines the relevance of *in vivo* assays with the simplicity and versatility of *in vitro* assays. In larvae, access to the developing vasculature is straightforward thanks to fluorophore-tagged strains and the small size of the animals makes the use of high-throughput strategies possible. In adults, the tailfin is equally convenient as a model tissue as regenerating vessels are directly observable at all stages and the animals are suitable for experimental manipulation with compounds, for instance. The advent of new genome modification techniques opens up even more tools for the vascular biologist as new therapeutic targets can be identified through mutational analysis.

## Author contributions

MC, GA, FF, MA, and TE all contributed to the conception of this manuscript, as well as to the acquisition and critical analysis of the intellectual content reviewed in this work. MC and GA were responsible for generating the images shown in Figure 1. The authors declare no potential conflict of interest, and approve this manuscript as the final version to be published.

## Funding

This work was partially financed by ICGEB (CRP/CHI11-01), FONDECYT Postdoctorado 2016 (Nr. 3160086) to MC, and the FONDAP Center for Genome Regulation (Nr. 15090007) to both MA and JE. The publication of this work was supported by the German Research Foundation (DFG) and the Technische Universität München within the funding program Open Access Publishing.

### Conflict of interest statement

The authors declare that the research was conducted in the absence of any commercial or financial relationships that could be construed as a potential conflict of interest.

## References

[B1] Akhtar N. Dickerson E. B. Auerbach R. (2002). The sponge/matrigel angiogenesis assay. Angiogenesis 5, 75–80. 10.1023/A:1021507031486 12549862

[B2] Albini A. Benelli R. (2007). The chemoinvasion assay: a method to assess tumor and endothelial cell invasion and its modulation. Nat. Protoc. 2, 504–511. 10.1038/nprot.2006.466 17406614

[B3] Arnaoutova I. George J. Kleinman H. K. Benton G. (2009). The endothelial cell tube formation assay on basement membrane turns 20: state of the science and the art. Angiogenesis 12, 267–274. 10.1007/s10456-009-9146-4 19399631

[B4] Arnaoutova I. Kleinman H. K. (2010). *In vitro* angiogenesis: endothelial cell tube formation on gelled basement membrane extract. Nat. Protoc. 5, 628–635. 10.1038/nprot.2010.6 20224563

[B5] Asnani A. Peterson R. T. (2014). The zebrafish as a tool to identify novel therapies for human cardiovascular disease. Dis. Model. Mech. 7, 763–767. 10.1242/dmm.016170 24973746 PMC4073266

[B6] Avraham-Davidi I. Ely Y. Pham V. N. Castranova D. Grunspan M. Malkinson G.. (2012). ApoB-containing lipoproteins regulate angiogenesis by modulating expression of VEGF receptor 1. Nat. Med. 18, 967–973. 10.1038/nm.2759 22581286 PMC3959651

[B7] Ayata R. Chabaud S. Auger M. Pouliot R. (2015). Behaviour of endothelial cells in a tridimensional *in vitro* environment. Biomed Res. Int. 2015:630461. 10.1155/2015/630461 25789323 PMC4350961

[B8] Azevedo A. Grotek B. Jacinto A. Weidinger G. Saúde L. Karl M. (2011). The regenerative capacity of the zebrafish caudal fin is not affected by repeated amputations. PLoS ONE 6:e22820. 10.1371/journal.pone.0022820 21829525 PMC3145768

[B9] Bagatto B. Francl J. Liu B. Liu Q. (2006). Cadherin2 (N-cadherin) plays an essential role in zebrafish cardiovascular development. BMC Dev. Biol. 6:23. 10.1186/1471-213X-6-23 16719917 PMC1523202

[B10] Baker M. Robinson S. D. Lechertier T. Barber P. R. Tavora B. D'Amico G.. (2011). Use of the mouse aortic ring assay to study angiogenesis. Nat. Protoc. 7, 89–104. 10.1038/nprot.2011.435 22193302

[B11] Baldessari D. Mione M. (2008). How to create the vascular tree? (Latest) help from the zebrafish. Pharmacol. Ther. 118, 206–230. 10.1016/j.pharmthera.2008.02.010 18439684

[B12] Bayliss P. E. Bellavance K. L. Whitehead G. G. Abrams J. M. Aegerter S. Robbins H. S.. (2006). Chemical modulation of receptor signaling inhibits regenerative angiogenesis in adult zebrafish. Nat. Chem. Biol. 2, 265–273. 10.1038/nchembio778 16565716 PMC1534118

[B13] Bedell V. M. Wang Y. Campbell J. M. Poshusta T. L. Starker C. G. Krug R. G.. (2012). *In vivo* genome editing using a high-efficiency TALEN system. Nature 491, 114–118. 10.1038/nature11537 23000899 PMC3491146

[B14] Benton G. Arnaoutova I. George J. Kleinman H. K. Koblinski J. (2014). Matrigel: from discovery and ECM mimicry to assays and models for cancer research. Adv. Drug Deliv. Rev. 79–80, 3–18. 10.1016/j.addr.2014.06.005 24997339

[B15] Berthod F. Symes J. Tremblay N. Medin J. Auger F. (2012). Spontaneous fibroblast−derived pericyte recruitment in a human tissue−engineered angiogenesis model *in vitro*. J. Cell. Physiol. 227, 2130–2137. 10.1002/jcp.22943 21769871

[B16] Bishop E. T. Bell G. T. Bloor S. Broom I. J. Hendry N. F. Wheatley D. N. (1999). An *in vitro* model of angiogenesis: basic features. Angiogenesis 3, 335–344. 10.1023/A:1026546219962 14517413

[B17] Boncler M. Różalski M. Krajewska U. Podsȩdek A. Watala C. (2014). Comparison of PrestoBlue and MTT assays of cellular viability in the assessment of anti-proliferative effects of plant extracts on human endothelial cells. J. Pharmacol. Toxicol. Methods 69, 9–16. 10.1016/j.vascn.2013.09.003 24103906

[B18] Boyden S. (1962). The chemotactic effect of mixtures of antibody and antigen on polymorphonuclear leucocytes. J. Exp. Med. 115, 453–466. 10.1084/jem.115.3.453 13872176 PMC2137509

[B19] Brown L. A. Rodaway A. R. Schilling T. F. Jowett T. Ingham P. W. Patient R. K.. (2000). Insights into early vasculogenesis revealed by expression of the ETS-domain transcription factor Fli-1 in wild-type and mutant zebrafish embryos. Mech. Dev. 90, 237–252. 10.1016/S0925-4773(99)00256-7 10640707

[B20] Bussmann J. Schulte-Merker S. (2011). Rapid BAC selection for tol2-mediated transgenesis in zebrafish. Development 138, 4327–4332. 10.1242/dev.068080 21865323

[B21] Bussmann J. Wolfe S. A. Siekmann A. F. (2011). Arterial-venous network formation during brain vascularization involves hemodynamic regulation of chemokine signaling. Development 138, 1717–1726. 10.1242/dev.059881 21429983 PMC3074448

[B22] Chávez M. N. Schenck T. L. Hopfner U. Centeno-Cerdas C. Somlai-Schweiger I. Schwarz C.. (2016). Towards autotrophic tissue engineering: photosynthetic gene therapy for regeneration. Biomaterials 75, 25–36. 10.1016/j.biomaterials.2015.10.014 26474040

[B23] Chen J. N. Haffter P. Odenthal J. Vogelsang E. Brand M. Eeden F. J.. (1996). Mutations affecting the cardiovascular system and other internal organs in zebrafish. Development 123, 293–302. 9007249 10.1242/dev.123.1.293

[B24] Childs S. Chen J.-N. N. Garrity D. M. Fishman M. C. (2002). Patterning of angiogenesis in the zebrafish embryo. Development 129, 973–982. 11861480 10.1242/dev.129.4.973

[B25] Choe C. P. Crump J. G. (2015). Eph-Pak2a signaling regulates branching of the pharyngeal endoderm by inhibiting late-stage epithelial dynamics. Development 142, 1089–1094. 10.1242/dev.115774 25725065 PMC4360181

[B26] Chung S. Sudo R. Vickerman V. Zervantonakis I. K. Kamm R. D. (2010). Microfluidic platforms for studies of angiogenesis, cell migration, and cell-cell interactions. Sixth International Bio-Fluid Mechanics Symposium and Workshop March 28-30, 2008 Pasadena, California. Ann. Biomed. Eng. 38, 1164–1177. 10.1007/s10439-010-9899-3 20336839

[B27] Chwalek K. Bray L. Werner C. (2014). Tissue-engineered 3D tumor angiogenesis models: potential technologies for anti-cancer drug discovery. Adv. Drug Deliv. Rev. 79–80, 3039. 10.1016/j.addr.2014.05.006 24819220

[B28] Cimpean A.-M. Ribatti D. Raica M. (2011). A brief history of angiogenesis assays. Int. J. Dev. Biol. 55, 377382. 10.1387/ijdb.103215ac 21858762

[B29] Clark E. R. Hitschler W. J. Kirby-Smith H. T. Rex R. O. Smith J. H. (1931). General observations on the ingrowth of new blood vessels into standardized chambers in the rabbit's ear, and the subsequent changes in the newly grown vessels over a period of months. Anat. Rec. 50, 29–167. 10.1002/ar.1090500203

[B30] Coffindaffer-Wilson M. Craig M. P. Hove J. R. (2011). Determination of lymphatic vascular identity and developmental timecourse in zebrafish (*Danio rerio*). Lymphology 44, 1–12. 21667817

[B31] Coomber B. L. Gotlieb A. I. (1990). *In vitro* endothelial wound repair. Interaction of cell migration and proliferation. Arteriosclerosis 10, 215–222. 10.1161/01.ATV.10.2.215 1969263

[B32] Couffinhal T. Silver M. Zheng L. P. Kearney M. Witzenbichler B. Isner J. M. (1998). Mouse model of angiogenesis. Am. J. Pathol. 152, 1667–1679. 9626071 PMC1858441

[B33] Coultas L. Chawengsaksophak K. Rossant J. (2006). Endothelial cells and VEGF in vascular development. Nature 438, 937–945. 10.1038/nature04479 16355211

[B34] Covassin L. D. Siekmann A. F. Kacergis M. C. Laver E. Moore J. C. Villefranc J. A.. (2009). A genetic screen for vascular mutants in zebrafish reveals dynamic roles for Vegf/Plcg1 signaling during artery development. Dev. Biol. 329, 212–226. 10.1016/j.ydbio.2009.02.031 19269286 PMC2791107

[B35] Cross L. M. Cook M. A. Lin S. Chen J.-N. N. Rubinstein A. L. (2003). Rapid analysis of angiogenesis drugs in a live fluorescent zebrafish assay. Arterioscler. Thromb. Vasc. Biol. 23, 911–912. 10.1161/01.ATV.0000068685.72914.7E 12740225

[B36] Diaz-Santana A. Shan M. Stroock A. D. (2015). Endothelial cell dynamics during anastomosis *in vitro*. Integr. Biol. 7, 454–466. 10.1039/C5IB00052A 25790315 PMC4515349

[B37] Donovan D. Brown N. J. Bishop E. T. Lewis C. E. (2001). Comparison of three *in vitro* human “angiogenesis” assays with capillaries formed *in vivo*. Angiogenesis 4, 113–121. 10.1023/A:1012218401036 11806243

[B38] Egaña J. Condurache A. Lohmeyer J. Kremer M. Stöckelhuber B. Lavandero S.. (2008). *Ex vivo* method to visualize and quantify vascular networks in native and tissue engineered skin. Langenbeck's Arch. Surg. 394, 349–356. 10.1007/s00423-008-0333-3 18458938

[B39] Ellertsdóttir E. Lenard A. Blum Y. Krudewig A. Herwig L. Affolter M.. (2010). Vascular morphogenesis in the zebrafish embryo. Dev. Biol. 341, 56–65. 10.1016/j.ydbio.2009.10.035 19895803

[B40] Fierro F. A. Kalomoiris S. Sondergaard C. S. Nolta J. A. (2011). Effects on proliferation and differentiation of multipotent bone marrow stromal cells engineered to express growth factors for combined cell and gene therapy. Stem Cells 29, 1727–1737. 10.1002/stem.720 21898687 PMC3784258

[B41] Gaiano N. Amsterdam A. Kawakami K. Allende M. Becker T. Hopkins N. (1996). Insertional mutagenesis and rapid cloning of essential genes in zebrafish. Nature 383, 829–832. 10.1038/383829a0 8893009

[B42] Gering M. Rodaway A. R. Göttgens B. Patient R. K. Green A. R. (1998). The SCL gene specifies haemangioblast development from early mesoderm. EMBO J. 17, 4029–4045. 10.1093/emboj/17.14.4029 9670018 PMC1170736

[B43] Gimbrone M. A. Leapman S. B. Cotran R. S. Folkman J. (1973). Tumor angiogenesis: iris neovascularization at a distance from experimental intraocular tumors. J. Natl. Cancer Inst. 50, 219–228. 4692862 10.1093/jnci/50.1.219

[B44] Gjini E. Hekking L. H. Küchler A. Saharinen P. Wienholds E. Post J.-A. A.. (2011). Zebrafish Tie-2 shares a redundant role with Tie-1 in heart development and regulates vessel integrity. Dis. Models Mech. 4, 57–66. 10.1242/dmm.005033 21045210 PMC3014345

[B45] Goodwin A. M. (2007). *In vitro* assays of angiogenesis for assessment of angiogenic and anti-angiogenic agents. Microvasc. Res. 74, 172–183. 10.1016/j.mvr.2007.05.006 17631914 PMC2692317

[B46] Gordon K. Schulte D. Brice G. Simpson M. A. Roukens M. G. Impel A.. (2013). Mutation in vascular endothelial growth factor-C, a ligand for vascular endothelial growth factor receptor-3, is associated with autosomal dominant milroy-like primary lymphedema. Circ. Res. 112, 956–960. 10.1161/CIRCRESAHA.113.300350 23410910

[B47] Gore A. V. Monzo K. Cha Y. R. Pan W. Weinstein B. M. (2012). Vascular development in the zebrafish. Cold Spring Harb. Perspect. Med. 2:a006684. 10.1101/cshperspect.a006684 22553495 PMC3331685

[B48] Gough W. Hulkower K. I. Lynch R. McGlynn P. Uhlik M. Yan L.. (2011). A quantitative, facile, and high-throughput image-based cell migration method is a robust alternative to the scratch assay. J. Biomol. Screen. 16, 155–163. 10.1177/1087057110393340 21297103

[B49] Greenway F. L. Liu Z. Yu Y. Caruso M. K. Roberts A. T. Lyons J.. (2007). An assay to measure angiogenesis in human fat tissue. Obes. Surg. 17, 510–515. 10.1007/s11695-007-9089-z 17608264

[B50] Habeck H. Odenthal J. Walderich B. Maischein H. Schulte-Merker S. (2002). Analysis of a zebrafish VEGF receptor mutant reveals specific disruption of angiogenesis. Curr. Biol. 12, 1405–1412. 10.1016/S0960-9822(02)01044-8 12194822

[B51] Haffter P. Granato M. Brand M. Mullins M. C. Hammerschmidt M. Kane D. A.. (1996). The identification of genes with unique and essential functions in the development of the zebrafish, *Danio rerio*. Development 123, 1–36. 9007226 10.1242/dev.123.1.1

[B52] Haldi M. Ton C. Seng W. L. McGrath P. (2006). Human melanoma cells transplanted into zebrafish proliferate, migrate, produce melanin, form masses and stimulate angiogenesis in zebrafish. Angiogenesis 9, 139–151. 10.1007/s10456-006-9040-2 17051341

[B53] Harder Y. Schmauss D. Wettstein R. Egaña J. T. T. Weiss F. Weinzierl A.. (2014). Ischemic tissue injury in the dorsal skinfold chamber of the mouse: a skin flap model to investigate acute persistent ischemia. J. Vis. Exp. 93:e51900. 10.3791/51900 25489743 PMC4354003

[B54] Hasan S. S. Siekmann A. F. (2015). The same but different: signaling pathways in control of endothelial cell migration. Curr. Opin. Cell Biol. 36, 86–92. 10.1016/j.ceb.2015.07.009 26241634

[B55] Heicklen-Klein A. Evans T. (2004). T-box binding sites are required for activity of a cardiac GATA-4 enhancer. Dev. Biol. 267, 490–504. 10.1016/j.ydbio.2003.09.042 15013808

[B56] Henkind P. (1978). Ocular neovascularization. The Krill memorial lecture. Am. J. Ophthalmol. 85, 287–301. 10.1016/S0002-9394(14)77719-0 580695

[B57] Hermkens D. M. Impel A. van Urasaki, A. Bussmann J. Duckers H. J. Schulte-Merker S. (2015). Sox7 controls arterial specification in conjunction with hey2 and efnb2 function. Development 142, 1695–1704. 10.1242/dev.117275 25834021

[B58] Herwig L. Blum Y. Krudewig A. Ellertsdottir E. Lenard A. Belting H.-G. G.. (2011). Distinct cellular mechanisms of blood vessel fusion in the zebrafish embryo. Curr. Biol. 21, 1942–1948. 10.1016/j.cub.2011.10.016 22079115

[B59] Hetheridge C. Mavria G. Mellor H. (2011). Uses of the *in vitro* endothelial-fibroblast organotypic co-culture assay in angiogenesis research. Biochem. Soc. Trans. 39, 1597–1600. 10.1042/BST20110738 22103493

[B60] Hill A. J. Teraoka H. Heideman W. Peterson R. E. (2005). Zebrafish as a model vertebrate for investigating chemical toxicity. Toxicol. Sci. 86, 6–19. 10.1093/toxsci/kfi110 15703261

[B61] Ho Y.-L. L. Lin Y.-H. H. Tsai I.-J. J. Hsieh F.-J. J. Tsai H.-J. J. (2007). *In vivo* assessment of cardiac morphology and function in heart-specific green fluorescent zebrafish. J. Formos. Med. Assoc. 106, 181–186. 10.1016/S0929-6646(09)60238-2 17389161

[B62] Hogan B. M. Herpers R. Witte M. Heloterä H. Alitalo K. Duckers H. J.. (2010). Vegfc/Flt4 signalling is suppressed by Dll4 in developing zebrafish intersegmental arteries. Development 136, 4001–4009. 10.1242/dev.039990 19906867

[B63] Huang C. C. Lawson N. D. Weinstein B. M. Johnson S. L. (2003). reg6 is required for branching morphogenesis during blood vessel regeneration in zebrafish caudal fins. Dev. Biol. 264, 263–274. 10.1016/j.ydbio.2003.08.016 14623247 PMC3665419

[B64] Huang H. Zhang B. Hartenstein P. A. Chen J. N. Lin S. (2005). NXT2 is required for embryonic heart development in zebrafish. BMC Dev. Biol. 5:7. 10.1186/1471-213X-5-7 15790397 PMC1079804

[B65] Hulkower K. I. Herber R. L. (2011). Cell migration and invasion assays as tools for drug discovery. Pharmaceutics 3, 107–124. 10.3390/pharmaceutics3010107 24310428 PMC3857040

[B66] Hwang W. Y. Fu Y. Reyon D. Maeder M. L. Tsai S. Q. Sander J. D.. (2013). Efficient genome editing in zebrafish using a CRISPR-Cas system. Nat. Biotechnol. 31, 227–229. 10.1038/nbt.2501 23360964 PMC3686313

[B67] Ichioka S. Shibata M. Kosaki K. Sato Y. Harii K. Kamiya A. (1997). Effects of shear stress on wound-healing angiogenesis in the rabbit ear chamber. J. Surg. Res. 72, 29–35. 10.1006/jsre.1997.5170 9344711

[B68] Irvin M. W. Zijlstra A. Wikswo J. P. Pozzi A. (2014). Techniques and assays for the study of angiogenesis. Exp. Biol. Med. 239, 14761488. 10.1177/1535370214529386 24872440 PMC4216737

[B69] Isogai S. Horiguchi M. Weinstein B. M. (2001). The vascular anatomy of the developing zebrafish: an atlas of embryonic and early larval development. Dev. Biol. 230, 278–301. 10.1006/dbio.2000.9995 11161578

[B70] Isogai S. Lawson N. D. Torrealday S. Horiguchi M. Weinstein B. M. (2003). Angiogenic network formation in the developing vertebrate trunk. Development 130, 5281–5290. 10.1242/dev.00733 12954720

[B71] Jin S.-W. W. Beis D. Mitchell T. Chen J.-N. N. Stainier D. Y. (2006). Cellular and molecular analyses of vascular tube and lumen formation in zebrafish. Development 132, 5199–5209. 10.1242/dev.02087 16251212

[B72] Jin S.-W. W. Herzog W. Santoro M. M. Mitchell T. S. Frantsve J. Jungblut B.. (2007). A transgene-assisted genetic screen identifies essential regulators of vascular development in vertebrate embryos. Dev. Biol. 307, 29–42. 10.1016/j.ydbio.2007.03.526 17531218 PMC2695512

[B73] Kamei M. Isogai S. Pan W. Weinstein B. M. (2010). Imaging blood vessels in the zebrafish. Methods Cell Biol. 100, 27–54. 10.1016/B978-0-12-384892-5.00002-5 21111213

[B74] Kepp O. Galluzzi L. Lipinski M. Yuan J. Kroemer G. (2011). Cell death assays for drug discovery. Nat. Rev. Drug Discov. 10, 221–237. 10.1038/nrd3373 21358741

[B75] Kimura E. Deguchi T. Kamei Y. Shoji W. Yuba S. Hitomi J. (2013). Application of infrared laser to the zebrafish vascular system: gene induction, tracing, and ablation of single endothelial cells. Arterioscler. Thromb. Vasc. Biol. 33, 1264–1270. 10.1161/ATVBAHA.112.300602 23539214

[B76] Kleaveland B. Zheng X. Liu J. J. Blum Y. Tung J. J. Zou Z.. (2009). Regulation of cardiovascular development and integrity by the heart of glass-cerebral cavernous malformation protein pathway. Nat. Med. 15, 169–176. 10.1038/nm.1918 19151727 PMC2665266

[B77] Köhler C. Orrenius S. Zhivotovsky B. (2002). Evaluation of caspase activity in apoptotic cells. J. Immunol. Methods 265, 97–110 10.1016/s0022-1759(02)00073-x 12072181

[B78] Kok F. O. Shin M. Ni C.-W. W. Gupta A. Grosse A. S. Impel A.. (2015). Reverse genetic screening reveals poor correlation between morpholino-induced and mutant phenotypes in zebrafish. Dev. Cell 32, 97–108. 10.1016/j.devcel.2014.11.018 25533206 PMC4487878

[B79] Küchler A. M. Gjini E. Peterson-Maduro J. Cancilla B. Wolburg H. Schulte-Merker S. (2006). Development of the zebrafish lymphatic system requires VEGFC signaling. Curr. Biol. 16, 1244–1248. 10.1016/j.cub.2006.05.026 16782017

[B80] Kunisaki Y. Frenette P. S. (2014). Influences of vascular niches on hematopoietic stem cell fate. Int. J. Hematol. 99, 699–705. 10.1007/s12185-014-1580-4 24756874

[B81] Lagendijk A. K. Yap A. S. Hogan B. M. (2014). Endothelial cell-cell adhesion during zebrafish vascular development. Cell Adh. Migr. 8, 136–145. 10.4161/cam.28229 24621476 PMC4049859

[B82] Larson E. M. Doughman D. J. Gregerson D. S. Obritsch W. F. (1997). A new, simple, nonradioactive, nontoxic *in vitro* assay to monitor corneal endothelial cell viability. Invest. Ophthalmol. Vis. Sci. 38, 1929–1933. 9331256

[B83] Lawson N. D. (2016). Reverse genetics in zebrafish: mutants, morphants, and moving forward. Trends Cell Biol. 2, 77–79. 10.1016/j.tcb.2015.11.005 26739910

[B84] Lawson N. D. Mugford J. W. Diamond B. A. Weinstein B. M. (2003). Phospholipase C gamma-1 is required downstream of vascular endothelial growth factor during arterial development. Genes Develop. 17, 1346–1351. 10.1101/gad.1072203 12782653 PMC196067

[B85] Lawson N. D. Scheer N. Pham V. N. Kim C. H. Chitnis A. B. Campos-Ortega J. A.. (2001). Notch signaling is required for arterial-venous differentiation during embryonic vascular development. Development 128, 3675–3683. 11585794 10.1242/dev.128.19.3675

[B86] Lawson N. D. Vogel A. M. Weinstein B. M. (2002). Sonic hedgehog and vascular endothelial growth factor act upstream of the Notch pathway during arterial endothelial differentiation. Dev. Cell 3, 127–136. 10.1016/S1534-5807(02)00198-3 12110173

[B87] Lawson N. D. Weinstein B. M. (2002a). *In vivo* imaging of embryonic vascular development using transgenic zebrafish. Dev. Biol. 248, 307–318. 10.1006/dbio.2002.0711 12167406

[B88] Lawson N. D. Weinstein B. M. (2002b). Arteries and veins: making a difference with zebrafish. Nat. Rev. Genet. 3, 674–682. 10.1038/nrg888 12209142

[B89] Lehr H. A. Leunig M. Menger M. D. Nolte D. Messmer K. (1993). Dorsal skinfold chamber technique for intravital microscopy in nude mice. Am. J. Pathol. 143, 1055–1062. 7692730 PMC1887078

[B90] Lenard A. Ellertsdottir E. Herwig L. Krudewig A. Sauteur L. Belting H.-G. G.. (2013). *In vivo* analysis reveals a highly stereotypic morphogenetic pathway of vascular anastomosis. Dev. Cell 25, 492–506. 10.1016/j.devcel.2013.05.010 23763948

[B91] Li J. Stuhlmann H. (2011). *In vitro* imaging of angiogenesis using embryonic stem cell-derived endothelial cells. Stem Cells Dev. 21, 331–342. 10.1089/scd.2010.0587 21385073 PMC3196834

[B92] Lieschke G. J. Currie P. D. (2007). Animal models of human disease: zebrafish swim into view. Nat. Rev. Genet. 8, 353–367. 10.1038/nrg2091 17440532

[B93] Limbourg A. Korff T. Napp L. C. Schaper W. Drexler H. Limbourg F. P. (2009). Evaluation of postnatal arteriogenesis and angiogenesis in a mouse model of hind-limb ischemia. Nat. Protoc. 4, 1737–1746. 10.1038/nprot.2009.185 19893509

[B94] Long Q. Meng A. Wang H. Jessen J. R. Farrell M. J. Lin S. (1997). GATA-1 expression pattern can be recapitulated in living transgenic zebrafish using GFP reporter gene. Development 124, 4105–4111. 9374406 10.1242/dev.124.20.4105

[B95] Mably J. D. Chuang L. P. Serluca F. C. Mohideen M. A. Chen J. N. Fishman M. C. (2006). santa and valentine pattern concentric growth of cardiac myocardium in the zebrafish. Development 133, 3139–3146. 10.1242/dev.02469 16873582

[B96] Mably J. D. Mohideen M. A. Burns C. G. Chen J.-N. N. Fishman M. C. (2003). heart of glass regulates the concentric growth of the heart in zebrafish. Curr. Biol. 13, 2138–2147. 10.1016/j.cub.2003.11.055 14680629

[B97] MacRae C. A. Peterson R. T. (2015). Zebrafish as tools for drug discovery. Nat. Rev. Drug Discov. 10, 721–731. 10.1038/nrd4627 26361349

[B98] Mayer F. L. Whalen E. A. Rheins L. A. (1994). A regulatory overview of alternatives to animal testing: United States, Europe, and Japan. J. Toxicol. Cutaneous Ocul. Toxicol. 13, 3–22. 10.3109/15569529409037506 11659896

[B99] Merfeld-Clauss S. Gollahalli N. March K. L. Traktuev D. O. (2010). Adipose tissue progenitor cells directly interact with endothelial cells to induce vascular network formation. Tissue Eng. A 16, 2953–2966. 10.1089/ten.tea.2009.0635 20486792 PMC2928048

[B100] Monti-Hughes A. Aromando R. F. Pérez M. A. Schwint A. E. Itoiz M. E. (2015). The hamster cheek pouch model for field cancerization studies. Periodontol. 2000 67, 292–311. 10.1111/prd.12066 25494606

[B101] Motoike T. Loughna S. Perens E. Roman B. L. Liao W. Chau T. C.. (2000). Universal GFP reporter for the study of vascular development. Genesis 28, 75–81. 10.1002/1526-968X(200010)28:2<75::AID-GENE50>3.0.CO;2-S 11064424

[B102] Moya-Díaz J. Peña O. A. Sánchez M. Ureta D. A. Reynaert N. G. Anguita-Salinas C.. (2014). Electroablation: a method for neurectomy and localized tissue injury. BMC Dev. Biol. 14:7. 10.1186/1471-213X-14-7 24528932 PMC3933190

[B103] Nasevicius A. Larson J. Ekker S. C. (2000). Distinct requirements for zebrafish angiogenesis revealed by a VEGF-A morphant. Yeast 17, 294–301. 10.1002/1097-0061(200012)17:4<294::AID-YEA54>3.0.CO;2-5 11119306 PMC2448381

[B104] Neufeld S. Planas-Paz L. Lammert E. (2014). Blood and lymphatic vascular tube formation in mouse. Seminars Cell Dev. Biol. 31, 115123. 10.1016/j.semcdb.2014.02.013 24631829

[B105] Nicosia R. F. (2009). The aortic ring model of angiogenesis: a quarter century of search and discovery. J. Cell. Mol. Med. 13, 4113–4136. 10.1111/j.1582-4934.2009.00891.x 19725916 PMC4496118

[B106] Nicosia R. Zorzi P. Ligresti G. Morishita A. Aplin A. (2011). Paracrine regulation of angiogenesis by different cell types in the aorta ring model. Int. J. Dev. Biol. 55, 447–453. 10.1387/ijdb.103222rn 21858770

[B107] Niles A. L. Moravec R. A. Eric Hesselberth P. Scurria M. A. Daily W. J. Riss T. L. (2007). A homogeneous assay to measure live and dead cells in the same sample by detecting different protease markers. Anal. Biochem. 366, 197–206. 10.1016/j.ab.2007.04.007 17512890

[B108] Niles A. L. Riss T. L. (2015). Multiplexed viability, cytotoxicity, and caspase activity assays. Methods Mol. Biol. 1219, 21–33. 10.1007/978-1-4939-1661-0_3 25308259

[B109] Norrby K. (2006). *In vivo* models of angiogenesis. J. Cell. Mol. Med. 10, 588–612. 10.1111/j.1582-4934.2006.tb00423.x 16989723 PMC3933145

[B110] Norrby K. C. (2011). Rat mesentery angiogenesis assay. J. Vis. Exp. 52:e3078. 10.3791/3078 21712799 PMC3121245

[B111] Palmer G. M. Fontanella A. N. Shan S. Hanna G. Zhang G. Fraser C. L.. (2011). *In vivo* optical molecular imaging and analysis in mice using dorsal window chamber models applied to hypoxia, vasculature and fluorescent reporters. Nat. Protoc. 6, 1355–1366. 10.1038/nprot.2011.349 21886101 PMC3500601

[B112] Pandya N. Dhalla N. Santani D. (2006). Angiogenesis—a new target for future therapy. Vascul. Pharmacol. 44, 265274. 10.1016/j.vph.2006.01.005 16545987

[B113] Pardo-Martin C. Chang T.-Y. Koo B. Gilleland C. Wasserman S. Yanik M. (2010). High-throughput *in vivo* vertebrate screening. Nat. Methods 7, 634–636. 10.1038/nmeth.1481 20639868 PMC2941625

[B114] Peterson R. T. Shaw S. Y. Peterson T. A. Milan D. J. Zhong T. P. Schreiber S. L.. (2004). Chemical suppression of a genetic mutation in a zebrafish model of aortic coarctation. Nat. Biotechnol. 22, 595–599. 10.1038/nbt963 15097998

[B115] Pfefferli C. Jaźwińska A. (2015). The art of fin regeneration in zebrafish. Regeneration 2, 72–83. 10.1002/reg2.33 PMC4895310 27499869

[B116] Poss K. D. Keating M. T. Nechiporuk A. (2003). Tales of regeneration in zebrafish. Dev. Dyn. 226, 202–210. 10.1002/dvdy.10220 12557199

[B117] Poujade M. Grasland-Mongrain E. Hertzog A. Jouanneau J. Chavrier P. Ladoux B.. (2007). Collective migration of an epithelial monolayer in response to a model wound. Proc. Natl. Acad. Sci. U.S.A. 104, 15988–15993. 10.1073/pnas.0705062104 17905871 PMC2042149

[B118] Qin L. Zeng H. Zhao D. (2006). Requirement of protein kinase D tyrosine phosphorylation for VEGF-A165-induced angiogenesis through its interaction and regulation of phospholipase Cgamma phosphorylation. J. Biol. Chem. 281, 32550–32558. 10.1074/jbc.M604853200 16891660

[B119] Raghunath M. Wong Y. Farooq M. Ge R. (2009). Pharmacologically induced angiogenesis in transgenic zebrafish. Biochem. Biophys. Res. Commun. 378, 766–771. 10.1016/j.bbrc.2008.11.127 19068208

[B120] Ramasamy S. K. Kusumbe A. P. Adams R. H. (2015). Regulation of tissue morphogenesis by endothelial cell-derived signals. Trends Cell Biol. 25, 148–157. 10.1016/j.tcb.2014.11.007 25529933 PMC4943524

[B121] Reed M. Damodarasamy M. Vernon R. (2011). Angiogenesis *in vitro* utilizing murine vascular explants in miniaturized 3-dimensional collagen gels. Open Circ. Vasc. J. 4, 12–17. 10.2174/1877382601104010012 24701258 PMC3972018

[B122] Rezzola S. Belleri M. Gariano G. Ribatti D. Costagliola C. Semeraro F.. (2014). *In vitro* and *ex vivo* retina angiogenesis assays. Angiogenesis 17, 429–442. 10.1007/s10456-013-9398-x 24121991

[B123] Ribatti D. (2008). Chick embryo chorioallantoic membrane as a useful tool to study angiogenesis. Int. Rev. Cell Mol. Biol. 270, 181–224. 10.1016/S1937-6448(08)01405-6 19081537

[B124] Ribatti D. Crivellato E. (2012). “Sprouting angiogenesis,” a reappraisal. Dev. Biol. 372, 157–165. 10.1016/j.ydbio.2012.09.018 23031691

[B125] Ribatti D. Nico B. Vacca A. Roncali L. Burri P. H. Djonov V. (2001). Chorioallantoic membrane capillary bed: a useful target for studying angiogenesis and anti-angiogenesis *in vivo*. Anat. Rec. 264, 317–324. 10.1002/ar.10021 11745087

[B126] Roman B. L. Pham V. N. Lawson N. D. Kulik M. Childs S. Lekven A. C.. (2002). Disruption of acvrl1 increases endothelial cell number in zebrafish cranial vessels. Development 129, 3009–3019. 12050147 10.1242/dev.129.12.3009

[B127] Sacilotto N. Monteiro R. Fritzsche M. Becker P. W. Sanchez-Del-Campo L. Liu K.. (2013). Analysis of Dll4 regulation reveals a combinatorial role for Sox and Notch in arterial development. Proc. Natl. Acad. Sci. U.S.A. 110, 11893–11898. 10.1073/pnas.1300805110 23818617 PMC3718163

[B128] Sarkanen J.-R. R. Vuorenpää H. Huttala O. Mannerström B. Kuokkanen H. Miettinen S.. (2012). Adipose stromal cell tubule network model provides a versatile tool for vascular research and tissue engineering. Cells Tissues Organs 196, 385–397. 10.1159/000336679 22739504

[B129] Schenck T. L. Chávez M. N. Condurache A. P. Hopfner U. Rezaeian F. Machens H.-G. G.. (2014). A full skin defect model to evaluate vascularization of biomaterials *in vivo*. J. Vis. Exp. 90:e51428. 10.3791/51428 25226211 PMC4828014

[B130] Schuermann A. Helker C. S. Herzog W. (2014). Angiogenesis in zebrafish. Semin. Cell Dev. Biol. 31, 106–114. 10.1016/j.semcdb.2014.04.037 24813365

[B131] Seano G. Chiaverina G. Gagliardi P. A. di Blasio L. Sessa R. Bussolino F.. (2013). Modeling human tumor angiogenesis in a three-dimensional culture system. Blood 121, e129–e137. 10.1182/blood-2012-08-452292 23471306

[B132] Shao Z. Friedlander M. Hurst C. Cui Z. Pei D. Evans L.. (2013). Choroid sprouting assay: an *ex vivo* model of microvascular angiogenesis. PLoS ONE 8:e69552. 10.1371/journal.pone.0069552 23922736 PMC3724908

[B133] Smith S. M. Wunder M. B. Norris D. A. Shellman Y. G. (2011). A simple protocol for using a LDH-based cytotoxicity assay to assess the effects of death and growth inhibition at the same time. PLoS ONE 6:e26908. 10.1371/journal.pone.0026908 22125603 PMC3219643

[B134] Sondergaard C. S. Hess D. A. Maxwell D. J. Weinheimer C. Rosová I. Creer M. H.. (2010). Human cord blood progenitors with high aldehyde dehydrogenase activity improve vascular density in a model of acute myocardial infarction. J. Transl. Med. 8:24. 10.1186/1479-5876-8-24 20214792 PMC2846892

[B135] Song H.-H. Park K. Gerecht S. (2014). Hydrogels to model 3D in vitro microenvironment of tumor vascularization. Adv. Drug Deliv. Rev. 79–80, 19–29. 10.1016/j.addr.2014.06.002 24969477 PMC4258430

[B136] Stainier D. Y. Weinstein B. M. Detrich H. W. Zon L. I. Fishman M. C. (1995). Cloche, an early acting zebrafish gene, is required by both the endothelial and hematopoietic lineages. Development 121, 3141–3150. 7588049 10.1242/dev.121.10.3141

[B137] Staton C. A. Reed M. W. Brown N. J. (2009). A critical analysis of current *in vitro* and *in vivo* angiogenesis assays. Int. J. Exp. Pathol. 90, 195–221. 10.1111/j.1365-2613.2008.00633.x 19563606 PMC2697546

[B138] Staton C. A. Stribbling S. M. Tazzyman S. Hughes R. Brown N. J. Lewis C. E. (2004). Current methods for assaying angiogenesis *in vitro* and *in vivo*. Int. J. Exp. Pathol. 85, 233–248. 10.1111/j.0959-9673.2004.00396.x 15379956 PMC2517524

[B139] Steinritz D. Schmidt A. Balszuweit F. Thiermann H. Ibrahim M. Bölck B.. (2015). Assessment of endothelial cell migration after exposure to toxic chemicals. J. Vis. Exp. 101:e52768. 10.3791/52768 26274775 PMC4544446

[B140] Stoddart M. J. (2011). Cell viability assays: introduction. Methods Mol. Biol. 740, 1–6. 10.1007/978-1-61779-108-6_1 21468961

[B141] Strilić B. Kucera T. Eglinger J. Hughes M. R. McNagny K. M. Tsukita S.. (2009). The molecular basis of vascular lumen formation in the developing mouse aorta. Dev. Cell 17, 505–515. 10.1016/j.devcel.2009.08.011 19853564

[B142] Styp-Rekowska B. Hlushchuk R. Pries A. R. Djonov V. (2011). Intussusceptive angiogenesis: pillars against the blood flow. Acta Physiol. 202, 213–223. 10.1111/j.1748-1716.2011.02321.x 21535415

[B143] Swift M. R. Pham V. N. Castranova D. Bell K. Poole R. J. Weinstein B. M. (2014). SoxF factors and Notch regulate nr2f2 gene expression during venous differentiation in zebrafish. Dev. Biol. 390, 116–125. 10.1016/j.ydbio.2014.03.018 24699544 PMC4104406

[B144] Tamplin O. Zon L. (2010). Fishing at the cellular level. Nat. Methods 7, 600–601. 10.1038/nmeth0810-600 20676080 PMC8933062

[B145] Taqueti V. R. Jaffer F. A. (2013). High-resolution molecular imaging via intravital microscopy: illuminating vascular biology *in vivo*. Integr. Biol. 5, 278–290. 10.1039/C2IB20194A 23135362 PMC3558556

[B146] Taylor K. Grant N. Temperley N. Patton E. (2010). Small molecule screening in zebrafish: an *in vivo* approach to identifying new chemical tools and drug leads. Cell Commun. Signal. 8:11. 10.1186/1478-811X-8-11 20540792 PMC2912314

[B147] Thisse B. Thisse C. (2014). *In situ* hybridization on whole-mount zebrafish embryos and young larvae. Methods Mol. Biol. 1211, 53–67. 10.1007/978-1-4939-1459-3_5 25218376

[B148] Tobia C. De Sena G. Presta M. (2011). Zebrafish embryo, a tool to study tumor angiogenesis. Int. J. Dev. Biol. 55, 505–509. 10.1387/ijdb.103238ct 21858773

[B149] Tran T. C. Sneed B. Haider J. Blavo D. White A. Aiyejorun T.. (2007). Automated, quantitative screening assay for antiangiogenic compounds using transgenic zebrafish. Cancer Res. 67, 11386–11392. 10.1158/0008-5472.CAN-07-3126 18056466

[B150] Traver D. Paw B. H. Poss K. D. Penberthy W. T. Lin S. Zon L. I. (2003). Transplantation and *in vivo* imaging of multilineage engraftment in zebrafish bloodless mutants. Nat. Immunol. 4, 1238–1246. 10.1038/ni1007 14608381

[B151] Ucuzian A. Greisler H. (2007). *In vitro* models of angiogenesis. World J. Surg. 31, 654–663. 10.1007/s00268-006-0763-4 17372665

[B152] Udvadia A. J. Linney E. (2003). Windows into development: historic, current, and future perspectives on transgenic zebrafish. Dev. Biol. 256, 1–17. 10.1016/S0012-1606(02)00083-0 12654288

[B153] Unger R. E. Krump-Konvalinkova V. Peters K. Kirkpatrick C. J. (2002). *In vitro* expression of the endothelial phenotype: comparative study of primary isolated cells and cell lines, including the novel cell line HPMEC-ST1.6R. Microvasc. Res. 64, 384–397. 10.1006/mvre.2002.2434 12453433

[B154] Vailhé B. Vittet D. Feige J. J. (2001). *In vitro* models of vasculogenesis and angiogenesis. Lab. Invest. 81, 439–452. 10.1038/labinvest.3780252 11304563

[B155] van Rooijen E. Voest E. E. Logister I. Korving J. Schwerte T. Schulte-Merker S.. (2009). Zebrafish mutants in the von Hippel-Lindau tumor suppressor display a hypoxic response and recapitulate key aspects of Chuvash polycythemia. Blood 113, 6449–6460. 10.1182/blood-2008-07-167890 19304954

[B156] Varshney G. K. Pei W. LaFave M. C. Idol J. Xu L. Gallardo V.. (2015). High-throughput gene targeting and phenotyping in zebrafish using CRISPR/Cas9. Genome Res. 25, 1030–1042. 10.1101/gr.186379.114 26048245 PMC4484386

[B157] Verseijden F. Posthumus-van Sluijs S. J. Pavljasevic P. Hofer S. O. Osch G. J. van Farrell E. (2010). Adult human bone marrow- and adipose tissue-derived stromal cells support the formation of prevascular-like structures from endothelial cells *in vitro*. Tissue Eng. A 16, 101–114. 10.1089/ten.tea.2009.0106 19642855

[B158] Wang P. Henning S. M. Heber D. (2010). Limitations of MTT and MTS-based assays for measurement of antiproliferative activity of green tea polyphenols. PLoS ONE 5:e10202. 10.1371/journal.pone.0010202 20419137 PMC2855713

[B159] Wang Y. Pan L. Moens C. B. Appel B. (2014). Notch3 establishes brain vascular integrity by regulating pericyte number. Development 141, 307–317. 10.1242/dev.096107 24306108 PMC3879812

[B160] Weiss A. Ding X. Beijnum J. Wong I. Wong T. Berndsen R.. (2015). Rapid optimization of drug combinations for the optimal angiostatic treatment of cancer. Angiogenesis. 18, 233–244. 10.1007/s10456-015-9462-9 25824484 PMC4473022

[B161] Weiss O. Kaufman R. Michaeli N. Inbal A. (2012). Abnormal vasculature interferes with optic fissure closure in lmo2 mutant zebrafish embryos. Dev. Biol. 369, 191–198. 10.1016/j.ydbio.2012.06.029 22819672

[B162] Whitfield M. L. George L. K. Grant G. D. Perou C. M. (2006). Common markers of proliferation. Nat. Rev. Cancer 6, 99–106. 10.1038/nrc1802 16491069

[B163] Wilkinson R. N. van Eeden F. J. (2014). The zebrafish as a model of vascular development and disease. Prog. Mol. Biol. Transl. Sci. 124, 93–122. 10.1016/B978-0-12-386930-2.00005-7 24751428

[B164] Xu C. Hasan S. S. Schmidt I. Rocha S. F. Pitulescu M. E. Bussmann J.. (2014). Arteries are formed by vein-derived endothelial tip cells. Nat. Commun. 5, 5758. 10.1038/ncomms6758 25502622 PMC4275597

[B165] Yang Y. Tang G. Yan J. Park B. Hoffman A. Tie G.. (2008). Cellular and molecular mechanism regulating blood flow recovery in acute versus gradual femoral artery occlusion are distinct in the mouse. J. Vasc. Surg. 48, 1546–1558. 10.1016/j.jvs.2008.07.063 19118738 PMC2791875

[B166] Yarrow J. C. Perlman Z. E. Westwood N. J. Mitchison T. J. (2004). A high-throughput cell migration assay using scratch wound healing, a comparison of image-based readout methods. BMC Biotechnol. 4:21. 10.1186/1472-6750-4-21 15357872 PMC521074

[B167] Young E. W. (2014). Advances in microfluidic cell culture systems for studying angiogenesis. J. Lab. Autom. 18, 427–436. 10.1177/2211068213495206 23832929

[B168] Yu J. A. Castranova D. Pham V. N. Weinstein B. M. (2015). Single-cell analysis of endothelial morphogenesis *in vivo*. Development 142, 2951–2961. 10.1242/dev.123174 26253401 PMC4582182

[B169] Zhen F. Lan Y. Yan B. Zhang W. Wen Z. (2013). Hemogenic endothelium specification and hematopoietic stem cell maintenance employ distinct Scl isoforms. Development 140, 3977–3985. 10.1242/dev.097071 24046317

[B170] Ziche M. Morbidelli L. (2015). The corneal pocket assay. Methods Mol. Biol. 1214, 15–28. 10.1007/978-1-4939-1462-3_2 25468596

